# The Prevalence of Gastrointestinal Disorders and Lifestyle Habits in Medical Students of Mashhad University of Medical Sciences: A Cross-Sectional Study

**DOI:** 10.34172/mejdd.3166

**Published:** 2026-01-31

**Authors:** Soroush Yazdani, Hanieh Salmani Izadi, Seyed Mohammad Matin Alavi Dana, Ali Okati, Shayan Yaghouti, Parto Pariz, Payam Sharifan, Zahra Gholamnezhad

**Affiliations:** ^1^Student Research Committee, Faculty of Medicine, Mashhad University of Medical Sciences, Mashhad, Iran; ^2^Applied Biomedical Research Center, Basic Sciences Research Institute, Mashhad University of Medical Sciences, Mashhad, Iran; ^3^International UNESCO Center for Health-Related Basic Sciences and Human Nutrition, Department of Nutrition, Faculty of Medicine, Mashhad University of Medical Sciences, Mashhad, Iran; ^4^Department of Physiology, Faculty of Medicine, Mashhad University of Medical Sciences, Mashhad, Iran

**Keywords:** Medical students, Gastrointestinal symptoms, Physical activity, Quality of life, Nutrition

## Abstract

**Introduction:**

This cross-sectional study aimed to assess the prevalence of gastrointestinal symptoms among medical students at Mashhad University of Medical Sciences (MUMS) and their association with lifestyle factors.

**Methods:**

Participants completed questionnaires evaluating gastrointestinal symptoms using the Gastrointestinal Symptom Rating Scale (GSRS), physical activity using the International Physical Activity Questionnaire (IPAQ), quality of life using 36-item short form health survey (SF-36), and dietary habits. Data were collected, categorized, and analyzed statistically.

**Results:**

Three hundred ninety-eight students were selected for analysis. Most participants had no complaints of reflux (62.8%), constipation (46%), or diarrhea (64.3%), or mild symptoms of abdominal pain (64.8%) and indigestion (85.9%). A total of 96.7% had mild or no symptoms according to GSRS. Male participants had higher reflux scores, while female participants reported more abdominal pain (*P*<0.01). No significant correlation was found between GSRS scores and lifestyle factors. Gastrointestinal symptoms were linked to reduced quality of life and mental/physical functioning (*P*<0.05, *P*<0.01). A negative correlation existed between SF-36 physical activity scores and participant sex (*P*<0.05).

**Conclusion:**

The study found a low prevalence of gastrointestinal symptoms among MUMS students. Digestive symptoms may affect quality of life physically and psychologically, but the severity of the symptoms may not be the main determinant of these effects.

## Introduction

 Gastrointestinal (GI) disorders include various conditions, including diarrhea and constipation, nausea, vomiting, belching, inflammatory bowel disease (IBD), irritable bowel syndrome (IBS), gastroesophageal reflux disease (GERD), and peptic ulcer, which affect many people worldwide. These disorders have a high prevalence and significantly reduce the quality of life in involved patients. Functional gastrointestinal disorders (FGIDs) were involved in more than 40% of people.^1^ According to Rome IV criteria, 25% of individuals aged 4-18 suffer from symptoms related to FGIDs.^2^ Due to its high prevalence, the economic burden of these disorders is also considerable.^3^ The high prevalence of functional bowel disorders, which is associated with low quality of life and productivity in patients, has increased the economic burden of the disease in Iran, too.^4^

 Despite a lack of sufficient evidence for beneficial preventive and therapeutic effects, many recommendations for a healthy lifestyle and diet have been made for the management of FGIDs. Recommendations such as avoiding and limiting the intake of alcohol, spicy foods, caffeine, fat, and insoluble fibers, as well as adhering to a regular daily eating pattern and physical activity, and ensuring enough hydration might be effective in relieving patients’ symptoms.^5^ Physical activity can be regarded as an important effector of the GI tract disorders. Several studies evaluated the beneficial impact of physical activity on different types of gastrointestinal disorders, such as peptic ulcers, and can decrease the risk of colon cancer.^6,7^ It is also shown that physical activity has an anti-inflammatory effect and might be regarded as a therapeutic and preventive strategy in IBD.^8^ Moreover, sedentary lifestyle is associated with obesity and increase in body mass index (BMI). In a cross-sectional study with 366 participants, the relationship between BMI and IBS was shown.^9^

 Change in other lifestyle components, like dietary habits, is recommended as a main therapeutic strategy in the management of GI disease. Avoiding caffeine, alcohol, large-volume meals, late meals, fat and spicy foods, and adhering to a low fermentable oligosaccharides, disaccharides, monosaccharides, and polyols (FODAMP) diet, regular meals, and good hydration are recommended for the management of IBS and GERD.^5,10^ However, more evidence is needed to propose standard dietary recommendations for GI disorders.

 To the best of our knowledge, there is no literature or cohort study on the prevalence of GI disorders in the adult population of Iran. Most studies have evaluated the prevalence of one FGID, such as IBS or GERD, in the population of Iranian university students.^11,12^ In addition, the correlation between lifestyle parameters and GI disease prevalence is not fully understood yet. Therefore, this study aimed to discover the prevalence of GI disorders and their relationship to quality of life, physical activity, and dietary habits among medical students.

## Methods

###  Study Subjects

 This cross-sectional study was conducted from September 2020 to July 2021 with the participation of medical students in Mashhad, Khorasan Razavi province, Iran. Medical students at Mashhad University of Medical Sciences (MUMS), aged 18-34 years, were informed about the aim and importance of the study, and 500 volunteers were recruited. No similar study has examined the prevalence of GI symptoms and their association with lifestyle among Iranian medical students. Consequently, the sample size for this study was calculated based on the prevalence of hard stools reported in the research conducted by Semnani et al.,^13^ using the formula for estimating a population proportion. Volunteers signed a consent form and were asked to complete a self-administered checklist and a form about demographic information, GI symptoms, quality of life, physical activity, and diet. Participants with the following features were excluded: incomplete responses, underlying conditions such as metabolic diseases, heart diseases, and diabetes mellitus, which may independently affect the quality of life and GI symptoms.

###  Questionnaires 

 Demographic information, including age, sex, weight, height, marital status, educational status, and history of illness or use of certain drugs, was collected via a checklist.

###  Questionnaire for Assessing the Severity of Gastrointestinal Symptoms 

 The Gastrointestinal Symptom Rating Scale (GSRS), as a standard self-assessment instrument in patients and normal populations, was used to measure the severity of GI symptoms. GSRS includes 15 items that can assess five categories: reflux, abdominal pain, indigestion, diarrhea, and constipation.^14^ The GSRS is a 7-point Likert Scale, with scores ranging from 1 (no symptoms) to 7 (most severe).^15^ A Persian version of the GSRS questionnaire, with high reliability (Cronbach’s alpha 0.76 for patients with FGID and 0.8 for healthy adults) and validity^16^, was applied. In this study, scores were categorized as follows: 1 for “none,” 2-3 for “mild,” 4-5 for “moderate,” and 6-7 for “severe”.^15^

###  Weir and Sherburne Questionnaire 

 To assess the quality of life and health in participants, a self-report short form (Persian version) of the Weir and Sherburne quality of life questionnaire (SF-36 Health Survey) was applied. The Iranian version of the scale had acceptable reliability (Cronbach’s alpha 0.77-0.99) and validity (correlation coefficients ranging from 0.58 to 0.95).^17^ It has 36 statements and evaluates eight domains of physical functioning (PF), role functioning-physical (RP), bodily pain (BP), general health (GH), Energy/fatigue (EF), emotional well-being (EW), social functioning (SF), and role functioning emotional (RE). Also, SF-36 provides two general domains: (1) the total score of the physical component (PCS), which also evaluates the physical dimension of health, and (2) the total score of the mental component (MCS), which also evaluates the psychosocial dimension of health. A higher score means a better quality of life.^18^

###  Physical Activity Questionnaire

 In this study, the level of physical activity was evaluated by the short version of the International Physical Activity Questionnaire (IPAQ). This self-reported questionnaire collects information about types of physical activity during a week, and the validity and reliability of its Persian version has been confirmed.^19^

 Physical activity was categorized as low (sedentary), moderate, or high (vigorous). The amount of activity was measured by multiplying resting metabolic rate by minutes of weekly performance (METs-minute/week) according to the IPAQ protocol.^20^

###  Food Recording Chart 

 Participants were asked to complete a food records checklist for three days in a week (including one holiday and two working days) to obtain nutrient intakes and energy. Dietary data were converted to grams and entered into the Nutritionist 4 software (First Databank, San Bruno, CA, United States) using the United States Department of Agriculture (USDA) food composition table. The database was modified to include Iranian foods.

###  Statistical Analysis 

 In order to analyze the data obtained by the questionnaires, appropriate methods of descriptive statistics were used to determine the frequency, percentage, mean, and standard deviation of the data. All data analysis was undertaken using version 22 of the Statistical Package for Social Sciences software (SPSS Inc., IL, USA). For normally distributed variables (age and BMI) the t-test was used. The Mann–Whitney U test was used for continuous variables if they were not normally distributed. Statistical significance was determined when the *P *value was < 0.05.

## Results

###  Demographic Data 

 Five hundred medical students completed the questionnaires, and after screening the forms for exclusion criteria (chronic disease or incomplete responses), 398 students were included in the final analyses. The average age of the participants was 20.7 ± 1.8 years, and there was a relatively even split between male participants (54%) and female participants (46%). The mean BMI of the total sample of our study was 22.6 ± 3.6 kg/m^2^. Based on the results of the completed surveys, 155 (38.9%) students were categorized as having a low level of physical activity, and 150 (37.7%) and 93 (23.4%) students were categorized as having medium and severe levels of physical activity, respectively. The majority of the participants were second-year medical students (Table 1).

**Table 1:** Demographic characteristics

	**Participants** **(n=398)**	***P *** **value**^a^ **(male vs. female)**
Age (year) Total (Mean ± SD)	20.79 ± 1.81	NS
Female (Mean ± SD) Male (Mean ± SD)	20.73 ± 1.77 20.84 ± 1.86	
Sex Number (%)		
Female	183 (46%)	
Male	215 (54%)	
BMI (kg/m2)		NS
Total (Mean ± SD) Female (Mean ± SD) Male (Mean ± SD)	22.67 ± 3.55 21.95 ± 3.24 23.28 ± 3.69	
Marital status Number (%)		
Single	383 (96.2%)	
Married	15 (3.8%)	
Year of MBBs course Number (%)		
First	8 (2%)	
Second	194 (48.7%)	
Third	182 (45.7%)	
Fourth	14 (3.6%)	
Physical activity Number (%)		
Low	155 (38.9%)	
Medium	150 (37.7%)	
Severe	93 (23.4%)	

a, t-test; SD, Standard deviation; NS, Not significant; MBBs, Bachelor of Medicine and Bachelor of Surgery

###  The Results of the GSRS 

 Details of the GI symptoms distribution are shown in Figure 1. Most of the participants had no complaint of reflux (62.8%), constipation (46%), or diarrhea (64.3%), or had mild symptoms of abdominal pain (64.8%) and indigestion (85.9%). The total GSRS of 96.7 % of students was mild (78.9%), or they had no symptoms (17.8%).

**Figure 1:**
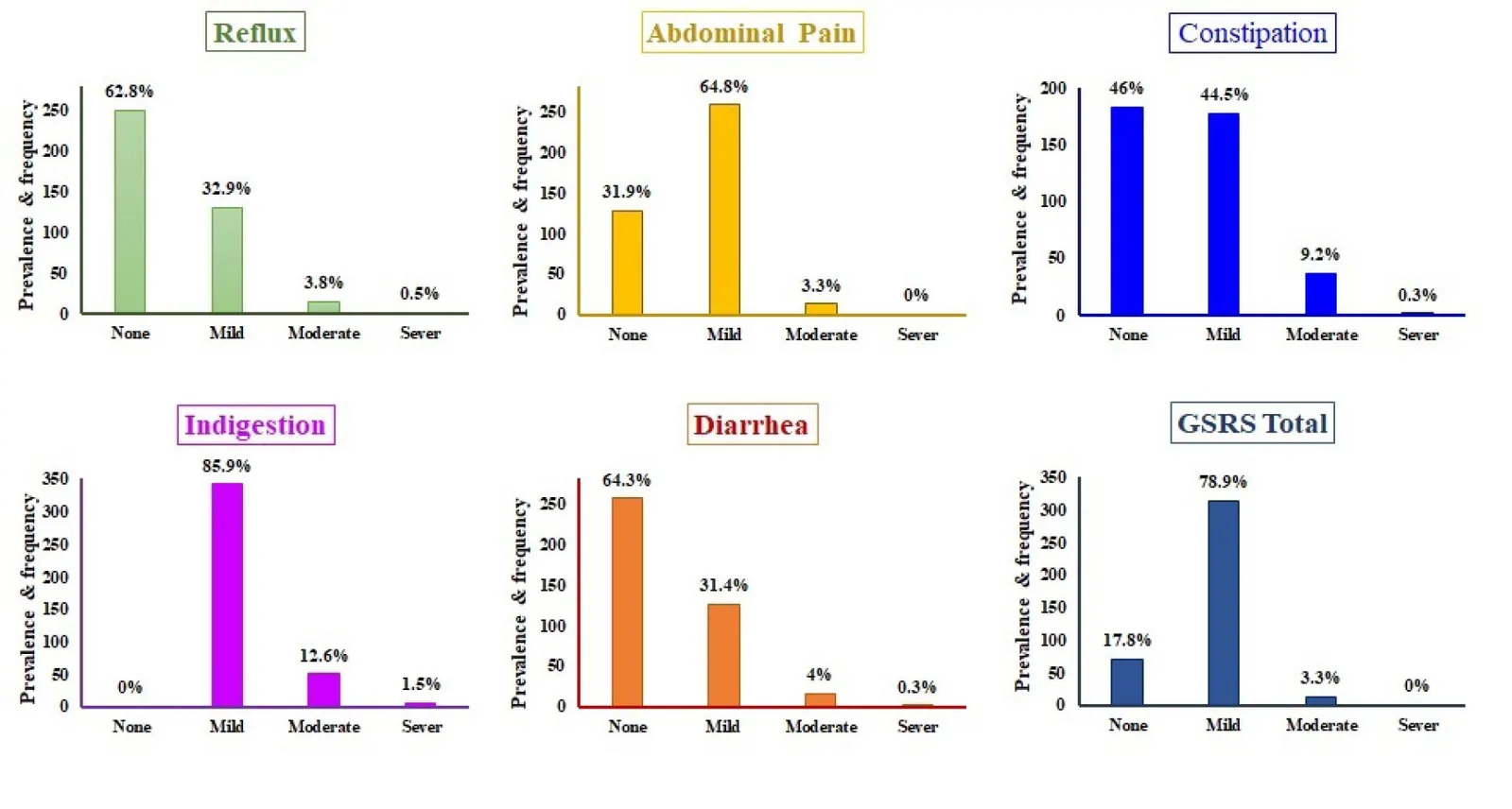
Distribution of Gastrointestinal symptoms (frequency and severity)

 The reflux syndrome score was higher in male students than in female students (P = 0.05), whereas the abdominal pain syndrome score was higher in female students than in male students (P < 0.001). There was no significant difference between the total GSRS score and the constipation, indigestion, and diarrhea syndromes in both sexes (Table 2).

**Table 2:** Gastrointestinal symptoms evaluated by the Gastrointestinal Symptom Rating Scale (GSRS)

	**Total**	**Male**	**Female**	***P *** **value**
Reflux	2.79 ± 1.51	2.81 ± 1.37	2.78 ± 1.66	0.05
Abdominal Pain	4.84 ± 2.09	4.60 ± 1.95	5.12 ± 2.21	< 0.001***
Constipation	4.91 ± 2.68	4.66 ± 2.29	5.21 ± 3.06	0.26
Indigestion	6.65 ± 2.99	6.51 ± 3.04	6.81 ± 2.93	0.07
Diarrhea	4.12 ± 2.19	4.32 ± 2.46	3.99 ± 1.81	0.26
GSRS Total	23.33 ± 7.72	22.92 ± 7.77	21.87 ± 7.65	0.11

Data are presented as Mean ± SD; Mann-Whitney (non-parametric). ***: Significant differences between male and female

###  Association of GSRS Scores and Physical Activity or Sex

 There was no correlation between total GSRS score, reflux, constipation, abdominal pain, indigestion, and diarrhea with physical activity (Table 3).

**Table 3:** Gastrointestinal Symptom Rating Scale (GSRS) scores and correlations with physical activity and sex

**Score**	**Mean (Minimum-Maximum)**	**Spearman's Correlation Coefficient **					
		**Physical activity**	**Reflux**	**AP**	**Con**	**Ind**	**Dia**
GSRS Total	23.33 (15-52)	0.017	0.652**	0.860**	0.800**	0.909**	0.685**
Reflux	2.79 (2-11)	0.023	1	0.618**	0.517**	0.530**	0.433**
AP	4.84 (3-15)	0.011	0.618**	1	0.670**	0.807**	0.604**
Con	4.91 (3-17)	-0.017	0.517**	0.670**	1	0.764**	0.414**
Ind	6.65 (4-19)	0.007	0.530**	0.807**	0.764**	1	0.619**
Dia	4.12 (3-17)	0.065	0.433**	0.604**	0.414**	0.619**	1

** Correlation is significant at the 0.01 level (2-tailed). AP, abdominal pain; Con, Constipation; Ind, Indigestion; Dia, Diarrhea

 In addition, there was a correlation between abdominal pain and sex (P < 0.01), while there was no association between other GSRS scores and sex. There was also a positive correlation across the different domains of GSRS (P < 0.01), indicating that higher scores in one domain were associated with higher scores in other domains, and vice versa.

###  The Results of the SF-36 Test

 The quality-of-life results, as assessed by both mental and physical functioning from the SF-36, were compared between groups by sex. The mental and physical components scores, as the eight health concepts from the SF-36, were compared and are summarized in Figure 2, where the lower the score is indicative of more impairment in quality of life. The mean quality-of-life scores for male and female students were 76.33 ± 10.86 and 73.55 ± 12.05, respectively. Again, a between-group comparison revealed that female participants had lower scores than male students on total quality of life, bodily pain, and mental and physical functioning (P < 0.05).

**Figure 2:**
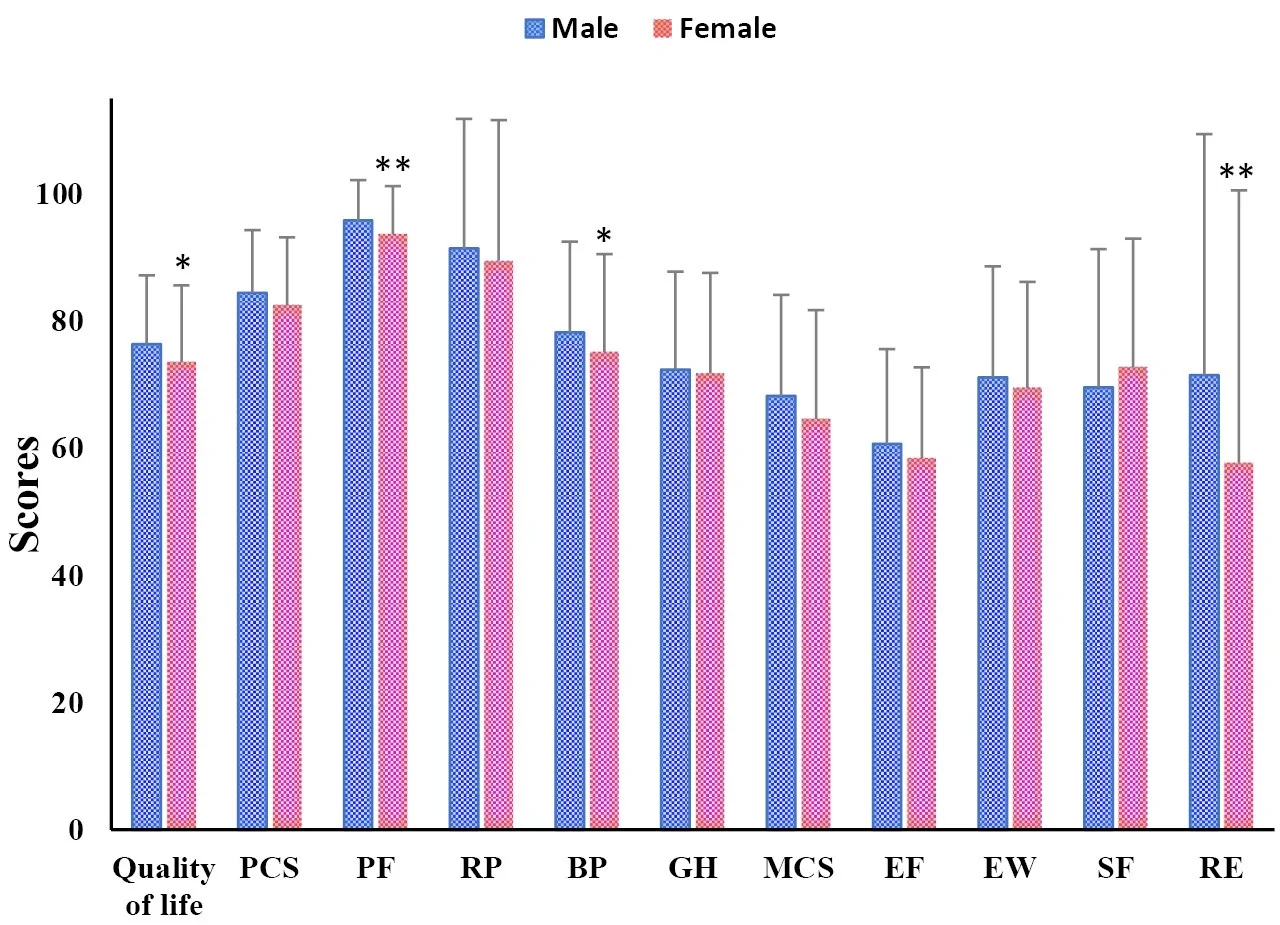
Distribution of health-related quality of life scores based on 36-item short form health survey (SF-36) domains in male and female medical students. PCS, physical component summary; PF, physical functioning; RP, role functioning-physical; BP, bodily pain; GH, general health; MCS, mental component summary; EW, emotional well-being; SF, social functioning; RE, role functioning emotional. Data are presented as Mean ± SD; Mann-Whitney (non-parametric). *: P < 0.05; **: P < 0.01, significant differences between male and female students

###  Associations of Quality of Life with GSRS Scores and Physical Activity 

 The correlations between quality of life (total, mental, and physical functioning) and GSRS and physical activity are presented in Tables 4 and 5. Having GI symptoms was significantly associated with lower quality of life, mental and physical functioning (*P* < 0.05, *P* < 0.01).

**Table 4:** Correlations of Gastrointestinal Symptom Rating Scale (GSRS) scores with quality of life domains

	**Spearman's Correlation Coefficient**		
	**Physical functioning**	**Mental functioning**	**Quality of life**
GSRS Total	-0.451**	-.353**	-0.441**
Reflux	-0.250**	-0.201**	-0.238**
AP	-0.4**	-0.282**	-0.358**
Con	-0.252**	-0.266**	-0.307**
Ind	-0.341**	-0.267**	-0.334*
Dia	-0.209**	-0.148**	-0.195**

** Correlation is significant at the 0.01 level (2-tailed). AP, abdominal pain; Con, Constipation; Ind, Indigestion; Dia, Diarrhea

**Table 5:** Correlations of quality of life domains with physical activity and sex

	**Spearman's Correlation Coefficient **	
	**Physical activity**	**Sex**
Physical functioning	0.070	-0.113*
Mental functioning	0.126*	-0.101*
Quality of life	0.114*	-0.117*

* Correlation is significant at the 0.05 level (2-tailed).

 Moreover, there was a negative correlation between the SF-36 main domain and physical activity and sex of participants (*P* < 0.05), except for the association of physical functioning with physical activity (Table 5).

###  Diet Record Macronutrient and GSRS Scores

 There was no association between the number of main meals, snacks, macronutrient intake (carbohydrate, protein, and fat), energy intake, and GSRS scores. However, there was a negative correlation between the number of snacks and abdominal pain and constipation (P < 0.05). Moreover, there was a positive association between diarrhea and fat and carbohydrate intake (P < 0.05). The details of the diet evaluation factors are summarized in Table 6.

**Table 6:** Correlations of diet with GSRS

	**Mean±SEM**	**rho, GSRS**	**rho, Reflux**	**rho, AP**	**rho, Con**	**rho, Ind**	**rho, Dia**
Main meal (N)	2.93 ± 0.03	-0.024	-0.048	-0.035	0.098	0.062	-0.093
Snack (N)	2.47 ± 0.09	-0.147	-0.015	-0.216*	-0.206*	-0.188	0.056
Carbohydrate (g)	425.73 ± 49.08	0.019	-0.021	0.008	-0.095	0.045	0.195*
Protein (g)	109.99 ± 14.04	-0.019	-0.007	0.015	-0.146	-0.033	0.071
Fat (g)	91.64 ± 7.54	0.070	0.012	0.072	-0.074	0.112	0.202*
Energy (Kcal)	2829.59 ± 286.65	0.064	0.008	0.068	-0.090	0.078	0.180

rho = Spearman correlation coefficient; AP: * Correlation is significant at the 0.05 level (2-tailed). AP, abdominal pain; Con, Constipation; Ind, Indigestion; Dia, Diarrhea

## Discussion

 According to the Rome Foundation Global Study, FGIDs are highly prevalent worldwide, with a significant burden on individuals and healthcare systems. The prevalence of these disorders varies across different regions, with higher rates in South America and Southeast Asia.^1^ FGIDs include diseases in which GI symptoms such as constipation, bloating, abdominal pain, and diarrhea are present without any specific problems in diagnostic tests. The organic cause of these diseases has not been identified, although communication disruption between the nervous and digestive systems has been proposed as the cause. Two important categories of these disorders include irritable bowel syndrome and functional dyspepsia.^21^ The results of this study showed a low prevalence of FGIDs symptoms in medical students of MUMS. Mild (85.9 %), moderate (12.6 %), and severe (1.5 %) symptoms of indigestion were the most common complaints of the different domains of GSRS scores, and their prevalence was higher in female students than in male students. There was a positive correlation among the different domains of GSRS scores, indicating a mutual association among symptoms of functional gastrointestinal disorders (FGIDs). Additionally, there is significant overlap between FGIDs and gastroesophageal reflux symptoms ^22^, suggesting that most FGIDs exhibit complex, multiple symptoms because of gut-brain axis disregulation.^21^ To the best of our knowledge, no study has evaluated the prevalence of FGIDs and the GSRS score among medical students in Iran. In the present study, more than 95% of subjects had mild or no symptoms of diarrhea or constipation, and there was no sex difference. However, there are different reports regarding the prevalence of GI disease in the previous studies. The IBS prevalence of Golestan University of Medical Sciences (n = 513) was evaluated by Semnani et al. based on the criteria of ROM II. About 10% of students reported symptoms of IBS, and the prevalence was higher in women.^13^ Among medical students at Gilan University (n = 422), the prevalence of IBS was 12.6% and was higher among female students.^23^ Lankarani et al. study showed that 16.4 of Shiraz University medical students (n = 801) had IBD based on Manning’s criteria.^24^ However, in the western region of Iran (Shahrekord), the one-year prevalence of IBS in 4762 adult subjects was lower (5.8%).^25^

 In the present study, 32.9% of students were scored as mild, 4.3% as moderate, and severe symptoms of reflux syndrome. Moreover, reflux syndrome scores were found to be higher in male students compared with female students. This finding is consistent with some previous studies suggesting a possible sex-dependent mechanism for gastroesophageal reflux disease (GERD). Several factors, including hormonal influences and physiological differences, may contribute to the observed variation in reflux symptoms between male and female students.^26^ A recent meta-analysis of 30 studies indicated Iran’s high prevalence of GERD symptoms. The overall, monthly, weekly, and daily prevalence in the adult population was 43.07%, 18.62%, 12.50%, and 5.64% respectively. In addition, the prevalence was higher in female (daily GERD = 7.88%) than in male individuals (daily GERD = 5.72%).^27^ Regarding medical students, there are a few studies. Bordbar et al. reported the GERD prevalence in medical students of Hormozgan University as 14.8% based on Montreal criteria.^28^ In another study, 25% of students at an Indian medical college had GERD, as assessed by a symptom frequency scale.^29^

 In this study, data on quality of life showed that female participants had lower scores in terms of total quality of life, bodily pain, and mental or physical functioning. which is consistent with a study in India.^15^ Some studies showed that, in general, male students had higher scores than female students in quality of life.^16,17^ In another study, it has been reported that there were no significant differences between male and female medical students according to quality of life.^18^ This discrepancy can be due to cultural and lifestyle differences.

 In addition, there was a negative correlation between all domains of GSRS scores and the main domain of quality of life, which shows a lower quality of life in subjects with higher GI symptoms. Reduction of quality of life has been reported in IBS and other functional intestinal and gastric symptoms.^30,31^ Neurasthenia and fatigue are more common in subjects with GI symptoms, and there is a strong association between mental (anxiety-related) and somatic dysfunction and GI disorders.^31^ In IBS patients, depressive symptoms, anxiety (GI-specific), and physical complaints are also important indicators of low quality of life.^30^ These effects might be explained by the brain-gut axis hypothesis. Possible factors such as gut microbiota, GI subtel inflammation, stress-induced elevation of corticotrophin­releasing hormone, and the close relation between the brain area that processes visceral afferents and the fear and depression controlling area (insula, amygdala, prefrontal cortex, and cingulate).^32^ Although GI symptoms are more common in women ^31^, we found no correlation between sex and the different domains of GSRS scores, except for abdominal pain, which was higher in female than male students.

 Total GSRS scores and other GSRS domains did not show a significant relationship with physical activity. This suggests that overall GI symptom severity, as measured by the GSRS, is not significantly affected by physical activity levels. However, the moderate intensity of regular exercise has been shown to attenuate the symptoms of FGIDs such as constipation, IBD, esophagitis, peptic ulcers, cholelithiasis, reflux, and colorectal cancer risk. Moreover, training mode, duration, and intensity, as well as medical monitoring of the patients’ symptoms and dietary intake, are important factors in the management of FGIDs by exercise.^6^

 In the present study, there was a negative correlation between the number of snacks and abdominal pain/constipation, as well as a positive association between diarrhea and fat/carbohydrate intake. In Iranian army men, it was demonstrated that a change in eating patterns, including slow food consumption (better chewing) and intake of breakfast, might help to reduce the prevalence of FGIDs and improve the quality of life.^33^ In addition to adhering to some diet recommendations, such as having meals regularly, reducing fat, spicy foods, and caffeine, as well as avoiding cigarettes and alcohol, might decrease the risk of FGIDs.^5,33^

 There are some limitations in the present study. The absence of a gold standard for precise diagnosis of GI diseases is the main limitation in the determination of FGIDS prevalence.^21^ In the present study, a validated self-administered GSRS questionnaire was used to determine the GI symptoms, but an interview-based Rome IV questionnaire seems to be more reliable. In addition, we used a daily food record, and factors inducing GI disorders, including alcohol, spicy food consumption, and cigarette smoking, were not evaluated in this study. Moreover, most of the participants were young and in the first year of their medical course, so no causal judgments can be drawn from the association data of this study, and future studies with a larger population are needed.

## Conclusion

 This study found a low prevalence of GI symptoms among medical students at MUMS. Although there was a negative association between GI symptoms and total quality of life, physical and mental functioning, there was no correlation between different GSRS domains and physical activity or macronutrient intake. It might be concluded that digestive disorders, both physically and psychologically, affect the quality of life, but symptom severity may not play a significant role in determining these effects.
